# Décrypter et caractériser les processus biologiques dynamiques qui rendent compte de la pérennité des populations de *Leishmania*

**DOI:** 10.48327/mtsi.v3i2.2023.384

**Published:** 2023-06-05

**Authors:** Émilie GIRAUD, Geneviève MILON

**Affiliations:** 1 Institut Pasteur, Université Paris Cité, Plateforme de criblage chémogénomique et biologique (PF-CCB), Centre de ressources et recherches technologiques (C2RT), UMR3523 Chimie biologique pour le vivant (Chem4Life); 2 Institut Pasteur, Université Paris Cité, Paris, France; * Actes du Colloque – Centenaire de la mort d'Alphonse Laveran. 24 novembre 2022, Paris / Proceedings of the Conference – Centenary of the death of Alphonse Laveran. 24 November 2022, Paris

**Keywords:** Alfonse Laveran, Edmond Sergent, Étienne Sergent, Paludisme, *Plasmodium*, *Leishmania*, Leishmaniose, Phlébotome, Hypersensibilité retardée, Algérie, Biskra, Maghreb, Afrique du Nord, Alfonse Laveran, Edmond Sergent, Étienne Sergent, Malaria, *Plasmodium*, *Leishmania*, Leishmaniasis, Sand fly, Delayed hypersensitivity, Algeria, Biskra, Maghreb, Northern Africa

## Abstract

Pour tenter de répondre le plus rigoureusement possible à cette question, il importait d'ancrer nos approches expérimentales aux observations et aux travaux pionniers de nos prédécesseurs, parmi lesquels Alphonse Laveran, Louis Parrot, Edmond et Étienne Sergent. Ces derniers, entre autres, avaient identifié comme populations naturellement hôtes de leishmanies, des rongeurs avec lesquels cohabitaient intimement des populations de phlébotomes hématophages telmophages.

Quand ont émergé les peuples d'humains au sein de tels écosystèmes naturels, à la sédentarisation d’*Homo sapiens,* des perturbations d'amplitude variable se seraient traduites par une transition de l'hématophagie de ces phlébotomes, de la zoophilie à la zooanthropophilie et à l'anthropophilie.

La création d'infrastructures où élever et intégrer dans des groupes expérimentaux, d'une part des phlébotomes holobiontes et, d'autre part des rongeurs de laboratoire holobiontes (rats, souris, hamsters, etc.) reste essentielle. Grâce à de telles infrastructures, il devient possible de saisir et caractériser les processus dynamiques multilatéraux – le plus souvent cliniquement silencieux – rendant compte de la biogenèse des niches tissulaires et/ou cellulaires protégeant des populations de morphotypes de *Leishmania* en développement, y compris ceux assurant la transmission d'hôte à hôte, bien qu'ils soient en petit nombre.

## Abstract (en)

To attempt resolving this issue accurately, it was necessary to anchor our experimental approaches in the observations and pioneering work of our predecessors, notably Alphonse Laveran, Louis Parrot, Edmond and Étienne Sergent. The latter, among other things, had identified as natural hosts of leishmaniasis, rodent populations with which hematophagous telmophagous sand fly populations cohabited closely.

When human populations emerged in these natural ecosystems, after the sedentarization of *Homo sapiens,* more or less important disturbances would have led to a transition of sand fly hematophagy, from zoophilia, to zoo-anthropophilia and anthropophilia.

The creation of infrastructures that allow the breeding and integration into experimental groups of both holobiont sand flies and holobiont laboratory rodents (rats, mice, hamsters, etc.) remains crucial. With such infrastructures, it becomes possible to grasp and characterize the multilateral dynamic processes – mostly clinically silent – that account for the biogenesis of tissue and/or cellular niches protecting populations of *Leishmania* developmental morphotypes, including those ensuring host-to-host transmission, albeit in small numbers.

Je remercie grandement les organisateurs et organisatrices de me faire bénéficier de cette très intéressante journée et de m'avoir permis de présenter cette intervention dont la préparation m'a procuré une grande émotion.^1^1Conférence prononcée par Geneviève Milon dont le texte a été transcrit à partir de son enregistrement par J.-P. Chippaux.

Mon intérêt pour les parasites intracellulaires découle de mon trajet d'exploratrice, curieuse scientifiquement, des macrophages, le système phagocytaire mononucléé qui a été si bien étudié par Elie Metchnikoff.

J'ai quelques petites remarques liminaires avant d'aborder le propos de cette présentation. Il faut souligner qu'Alphonse Laveran, en 1907, n'a pas qualifié les maladies sur lesquelles il travaillait parce que, à l’époque, il s'occupait de maladies humaines. Or, comme vous le savez, les humains sont les derniers à avoir émergé sur la planète Terre – on les appelle maintenant les hominidés^2^2Dont l'humain(*Homo sapiens*), est actuellement le seul représentant. –, ce qui les a amenés à interférer avec les écosystèmes naturels préexistants. Mais j'ai été rassurée de voir que, dans sa thèse de médecine soutenue le 29 novembre 1867 – une véritable thèse de science rapportant une expérimentation rigoureuse sur la régénération axonale confirmée par la récupération fonctionnelle [4] – Alphonse Laveran avait posé 3 exigences qui sont ancrées à son remarquable parcours de formation. La première est celle d'une observation histologique minutieuse. La deuxième est de se référer à la fonction pour expliquer les données structurales. Enfin, la troisième a trait à une interprétation méticuleuse de l'observation microscopique [1]. Monsieur Laveran était fondamentalement attentif à toutes les techniques disponibles à son époque et, je le souligne, le microscope a été pour lui très important pour comparer, d'une part les tissus d'individus sains décédés d'accident – des militaires le plus souvent – avec, d'autre part ceux de patients qui présentaient une fièvre palustre.

Un tableau du fils de George Sand qui vivait dans le Berry, *Le fantôme du marais* (Fig. 1), évoque Monsieur Laveran qui a pris la place du fantôme pour essayer de comprendre comment ces fièvres pouvaient émerger, d'abord et avant tout, de ce qui était à l’époque des marécages avec des eaux stagnantes et propres. L'idée de regarder une goutte de sang frais dans un microscope monoculaire éclairé par la lumière de l'Algérie, à une température qui n’était évidemment, ni celle de l'accès fébrile, ni la température normale d'un être sain, est pour moi lumineuse. Certes à l’époque, il n’était pas encore possible de colorer des frottis sanguins. Je voudrais revenir juste sur la fameuse planche représentant différents stades du *Plasmodium*, examinés au grossissement 400 [6]. Il avait fait la relation entre les corps ovalaires – correspondant au microgamétocyte qu'il représente d'ailleurs sans que l'on puisse voir la membrane du globule rouge, la cellule hôte, et au macrogamétocyte de taille plus grande – et ces fameuses ex-flagellations à partir d'un globule rouge qu'il observait (Fig. 2). En outre, il avait aussi vu à, partir de ponctions de rate réalisées pour identifier l’étiologie des fièvres lorsqu'il y avait une hépato-splénomégalie, les formes du parasite présentes dans la rate, sans pouvoir anticiper que cela pouvait être le siège de la gamétocytogenèse.

**Figure 1:**
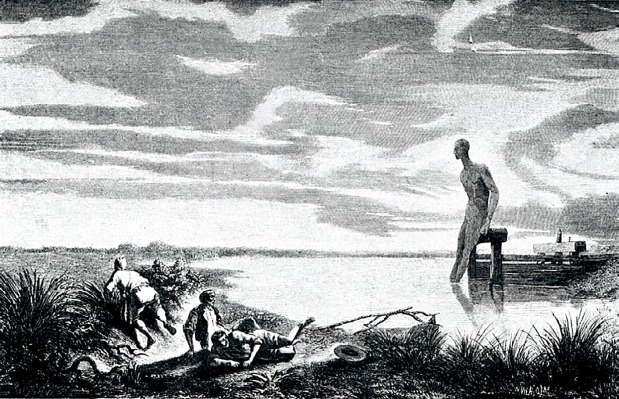
Le fantôme du marais. Une allégorie du paludisme. Maurice Dudevant, dit Maurice Sand (1823-1889) The ghost of the swamp. An allegory of malaria. Maurice Dudevant, known as Maurice Sand (1823-1889)

**Figure 2:**
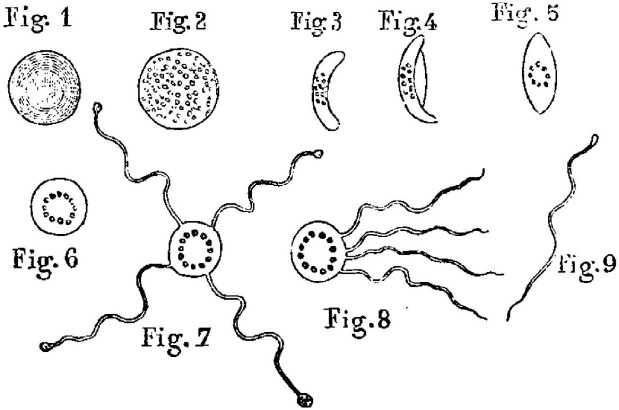
Dessin d'Alphonse Laveran représentant les formes sanguines de *Plasmodium vivax* [6] Plasmodium vivax *blood cell morphology drawn by Alphonse Laveran [6]*

En 1880, il a publié une observation de leishmaniose faite à l'hôpital militaire de Biskra avant de quitter l'Algérie pour revenir en France [5]. Il y expliquait que des êtres humains vivant dans un écosystème particulier en saison humide, présentaient des lésions cutanées sur la face ou sur les régions découvertes du corps. Ses deux complices, Edmond et Etienne Sergent, ont poursuivi cette recherche entre les années 1910 et 1920, sans doute grâce à Emile Roux, au moins par écrit, un médiateur extraordinaire. Ce dernier avait proposé à Edmond et Etienne Sergent qui voulaient analyser les anophèles en Algérie de créer une mission pour qu'ils puissent travailler sur le rôle réel des anophèles hématophages comme hôtes et vecteurs potentiels des formes transmissibles d'hôte à hôte. Cela a été compris ainsi, ce que je trouve remarquable parce que nous continuons à parler des maladies transmissibles vectorielles. On sait aujourd'hui que le vecteur doit être hématophage, du moins dans le monde des insectes, et posséder un ou plusieurs tissus où se développe un parasite eucaryote.

Quoiqu'il en soit, cette mission a été remarquablement fructueuse dépassant les attentes d'Emile Roux. J'ai été très intéressée que la première image d'un schéma qui a été fait à l'usage des enfants des écoles qualifiées de coloniales ou autochtones, associe, d'une part l'insecte hématophage – le phlébotome soulignant sa petite taille au point que Monsieur Laveran l'avait appelé le moucheron – avec ses différents stades de développement, et d'autre part, le parasite dans ses formes intra- et extracellulaires représentées dans le cartouche de la figure (Fig. 3) [7, 8].

**Figure 3:**
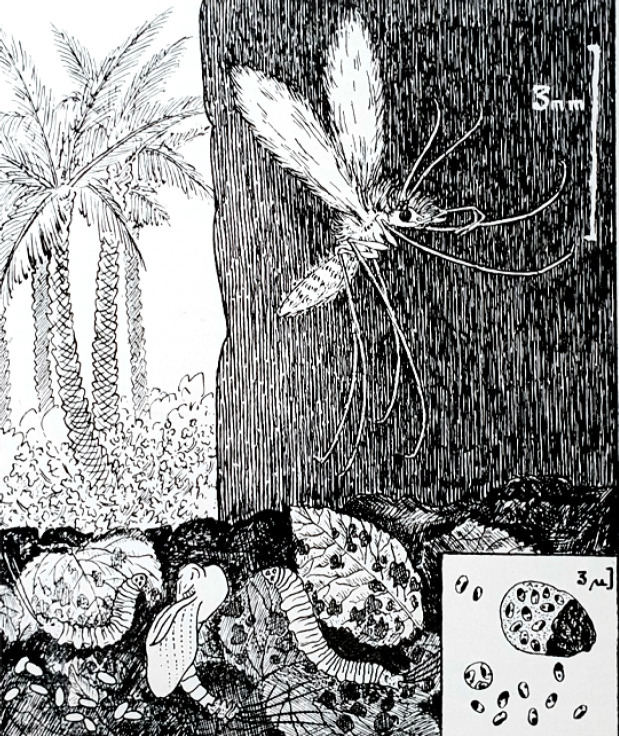
Dessin d'Edmond Sergent représentant *Phlebotomus papatasi* et ses larves telluriques; en cartouche, *Leishmania tropica* intra- et extracellulaire [7] Phlebotomus papatasi *and its telluric larvae; in cartouche, intra- and extracellular* Leishmania tropica. *Drawing by Edmond Sergent [7]*

Quand j'ai vu la taille de ces moucherons – les phlébotomes – je me suis posé la question de la possibilité de créer un modèle expérimental où on réaliserait, grâce à des méthodes de culture de leishmanies in vitro dont je n'avais pas appréhendé les limites, l’étude des processus rendant compte d'une lésion cutanée transitoire guérissant spontanément. Malheureusement, cela a été un échec parce que même si l'on enrichit le milieu mis au point par Charles Nicolle avec des techniques qui sont loin d’être idéales, nous n'obtenons jamais une population suffisante de promastigotes extracellulaires cultivés en condition axénique.

Si l'on veut vraiment étudier le développement du parasite sous-tendant sa pérennité et les processus induisant les lésions cutanées transitoires après leur réparation, il faut absolument mettre au point un modèle expérimental approprié. Il est maintenant bien établi qu'il existe du parasitisme asymptomatique dans les zones où il y a une population très abondante de phlébotomes qui cohabitent dans les galeries de rongeurs. Ils y trouvent les végétaux et les fèces nécessaires à leur alimentation et sortent au crépuscule pour leur repas sanguin afin d'assurer leur descendance, donc leur pérennité. En outre, un moucheron aussi petit peut difficilement aller prélever son repas sanguin sur les peaux recouvertes de fourrure des rongeurs. Les rares endroits où il n'y a pas de fourrure chez les hôtes naturels, ce sont le pavillon auriculaire et le museau. Ainsi, avons-nous eu recours au pavillon auriculaire de souris de laboratoire élevées dans des conditions gnotobiotiques, et à des phlébotomes élevés en insectarium pour observer ces deux mécanismes et les dissocier. A partir de ce modèle expérimental, il a été possible, d'analyser une autre particularité du repas sanguin des phlébotomes – la telmophagie – qui, comme leur nom le suggère, dilacèrent les micro-vaisseaux du derme vascularisé, et inoculent un peu de leur salive pour empêcher la coagulation et former une collection de sang qu'ils ingèrent. Lors de cette opération, ils délivrent cette population de parasites que nous étions incapables de produire *in vitro*, qui est une population sortie du cycle cellulaire dont l'effectif d'ailleurs n'est pas très élevé.

Ce modèle non naturel mais pertinent quant au site de piqûre qu'est le pavillon auriculaire, n'existe pas encore à l'Institut Pasteur. Il nécessite que soient construits des insectariums, où élever et intégrer dans les groupes expérimentaux des phlébotomes holobiontes, et des infrastructures où élever et intégrer dans ces mêmes groupes expérimentaux des souris de laboratoire consanguines holobiontes^3^3National Institute of Allergy and Infectious Diseases, institution gouvernementale des États-Unis.. Faute d'insectarium, Yasmine Belkaid a été accueillie par le NIH qui détient de telles infrastructures permettant l'analyse de la biologie du développement du parasite qui sous-tend sa pérennité à la fois chez les souris et les phlébotomes de laboratoire.

Cela lui a permis, ainsi qu’à Matthew Rogers et Paul Bates qui, en Angleterre, avaient aussi accès à des insectariums dans les fameuses écoles de médecine tropicale de Londres et de Liverpool, d'observer des phénomènes extrêmement importants [2]. La telmophagie a été très bien reproduite – toutefois, il ne s'agissait pas de l'action d'un seul phlébotome hôte de parasites mais de celles de 10 phlébotomes sur une souris anesthésiée – et vous voyez vraiment les traces des sites hémorragiques avec du sang non coagulé sous l'action de la salive. En outre, il a été montré, non pas au NIH mais en Angleterre, qu'il y avait un bouchon de gel dont il a été facile d’établir qu'il était généré par la forme du parasite qui proliférait lentement mais qui n’était pas encore sorti du cycle cellulaire. Outre la telmophagie naturelle, cela compliquait énormément le dépôt des parasites transmissibles dans le derme du pavillon auriculaire. On estime actuellement qu'il y a entre 1 et 10 parasites transmissibles capables de s’établir chez la souris de laboratoire de même que, probablement, chez le rongeur sauvage originel dont l’écosystème a été perturbé, pour ne pas dire en partie détruit, par *Homo sapiens* qui, du coup, est devenu hôte. On sait depuis peu, grâce aux travaux menés en Angleterre, que ce bouchon de gel parasitaire, d'abord analysé comme une agrégation des formes métacycliques, était en fait un site de métacytogenèse grâce à un manteau de lipophosphoglycanes. Peut-être, si un jour on sait reproduire ce processus in vitro, en apprendra-t-on encore un peu plus.

Outre le vecteur hôte de promastigotes métacycliques, les souris de laboratoire holobiontes présentent également un grand intérêt. Les processus immuns qui œuvrent au niveau du pavillon auriculaire où a été inoculée une population de parasites, quel que soit l'effectif, sont d'un tel dynamisme qu'il est difficile d'apprécier précisément toutes les interactions, sauf à imaginer des souris rapporteuses de toutes les cellules du système immunitaire local au niveau des tissus barrières.

Le macrophage du derme, dont l'ontogenèse provient du sac vitellin, est la cellule de longue durée de vie privilégiée où le métacyclique se transforme en amastigote intracellulaire au sein d'une vacuole, lequel n'est pas mobile bien que possédant une ébauche de poche flagellaire. Ensuite, au moment où les systèmes immunitaires proinflammatoires sont dominants, il y a un contrôle de la population des amastigotes qui ont comme cellules clés préférentielles des cellules dendritiques qui sont aussi des cellules phagocytaires immatures – la notion de maturité étant importante.

Nous n'avons pas encore réussi à établir si dans ces cellules, l'amastigote potentiellement transmissible se multiplie très lentement, comme le bradyzoïte du toxoplasme, ou s'il sort du cycle cellulaire et entre en phase G0. Les techniques actuelles d'imagerie dynamique favorisant la visualisation du parasite, des cellules hôtes et de leur renouvellement, ainsi que les techniques d'omiques permettront peut-être de l’établir. Quand le phlébotome prend un repas sanguin sur cette peau qui est, non seulement guérie mais où la forme transmissible persiste, les volumes de sang prélevés sont de l'ordre de quelques nanolitres. Le terme de moucheron que lui avait donné initialement Monsieur Laveran trouve ici tout son sens.

Je voudrais juste intégrer une dernière donnée qui me paraît extrêmement importante, à savoir qu’à Biskra, M. Laveran avait remarqué sur les peaux de patients sains qui vivaient dans les endroits où il y avait des clous de Biskra, une abondance de femelles hémato-phages prenant un repas sanguin, et de mâles. Il s’était interrogé sans pouvoir y répondre lui-même – mais les frères Sergent l'ont fait ensuite – sur la potentielle importance d'une immunisation qui serait strictement liée aux composants salivaires. Il en découle, puisque la fréquence des mouches hôtes de métacy-cliques est relativement faible, la question de savoir si, dans les écosystèmes comme ceux de Biskra et surtout dans ceux où se déploient les rongeurs, les repas sanguins pris par les phlébotomes pourraient déjà préparer la niche du parasite dermotrope.

José Ribeiro, toujours au NIH, a montré que la salive des phlébotomes libres de tout parasite était en mesure d'immuniser des souris de laboratoire indemnes à ce moment-là de leishmanies. Les antigènes de la salive inoculés strictement dans le derme provoqueraient une réponse inflammatoire physiologique régulée sous la forme d'une réaction d'hypersensibilité de type retardé [3]. Celle-ci exerce une protection contre l'infection par des leishmanies lors de piqûres ultérieures de phlébotomes hôtes de leishmanies métacycliques, grâce à l'augmentation du flux sanguin local et à la brièveté alors constatée du repas sanguin (Fig. 4) – dont on se souvient qu'il est compliqué à cause de la telmophagie –, ce qui favorise le développement d'un parasitisme asymptomatique.

**Figure 4:**
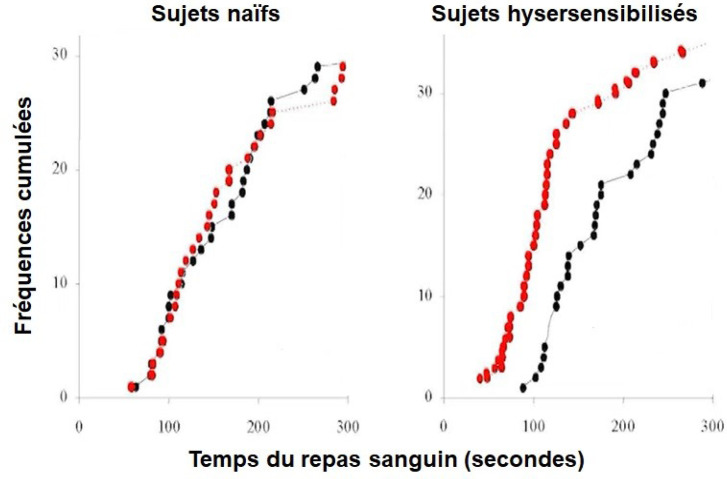
Temps d'alimentation de 32 *P. papatasi* (par ordre de classement des résultats cumulés) sur la face antérieure de l'avant-bras de 3 volontaires humains naïfs et 3 autres présentant une réaction d'hypersensibilité retardée à la piqûre de phlébotome. Les mesures ont été effectuées dans des sites cutanés normaux (cercles noirs) ou proches (5 mm) des sites cutanés où les phlébotomes s’étaient nourris la veille (cercles rouges) [3] *Feeding times of 32* P. papatasi *(rank order of the cumulative results) on the anterior forearm of 3 naive human volunteers and 3 others displaying delayed hypersensitivity reaction to sand fly bite. Measurements were made in normal skin sites (black dots) or near (5 mm) skin sites where sand flies had fed the day before (red dots) [3]*

Des vaccins reposant sur la salive des phlébotomes ont déjà été testés dans différents pays où se trouvent, d'ailleurs, d'autres espèces.

Pour conclure, je voudrais juste dire combien nous avions apprécié d'avoir des infrastructures pour les rongeurs mais qu'il va falloir aussi construire des vivariums pour analyser la coévolution des parasites protozoaires avec leur hôte insecte comme avec leur hôte bio-logiquement pertinent que sont les rongeurs.

## Liens D'intérêts

Les autrices ne déclarent aucun lien d'intérêt.
